# A new dynamic word learning task to diagnose language disorder in French-speaking monolingual and bilingual children

**DOI:** 10.3389/fresc.2022.1095023

**Published:** 2023-01-30

**Authors:** Mélodie Matrat, Hélène Delage, Margaret Kehoe

**Affiliations:** Psycholinguistics and Speech-Language Therapy, Faculty of Psychology and Educational Sciences, University of Geneva, Geneva, Switzerland

**Keywords:** word learning, shared storybook reading, developmental language disorder (DLD), dynamic assessment (DA), diagnostic accuracy, bilingualism, children

## Abstract

Tools to effectively assess the language performance of bilingual children are lacking. Static tests assessing vocabulary knowledge (e.g., naming task) are not appropriate for testing bilingual children due to different types of bias. Alternative methods have been developed to diagnose bilingual children, including measuring language learning (e.g., word learning) through dynamic assessment. Research conducted with English-speaking children indicates that DA of word learning is effective in diagnosing language disorders in bilingual children. In this study, we examine whether a dynamic word learning task, using shared-storybook reading, can differentiate French-speaking (monolingual and bilingual) children with developmental language disorder (DLD) from those with typical development (TD). Sixty children (4–8 years), 43 with TD and 17 with DLD, participated: 30 were monolinguals and 25 were bilinguals. The dynamic word-learning task used a shared-storybook reading context. The children had to learn four non-words, paired with novel objects, as well as their semantic characteristics (a category and a definition) during the reading of a story. Post-tests assessed the recall of the phonological form and the semantic features of the objects. Phonological and semantic prompts were given if the child was unable to name or describe the objects. Results indicated that children with DLD performed less well than those with TD on phonological recall, leading to fair sensitivity and good specificity at delayed post-test for young children (4–6 years). Semantic production did not differentiate the two groups: all children performed well at this task. In sum, children with DLD have more difficulties encoding the phonological form of the word. Our findings suggest that a dynamic word learning task using shared-storybook reading is a promising approach for diagnosing lexical difficulties in young French-speaking, monolingual and bilingual, children.

## 1. Introduction

In everyday life, having a large lexicon is important. Words form the basis of language: they link a phonological form and a referent with its semantic features (meaning) and this knowledge is shared by everyone to communicate. Moreover, vocabulary growth is important for achieving academic success (1, 2). It is well known that typically developing (TD) bilingual children exhibit different patterns of vocabulary development compared to monolingual children (e.g. (3)). Children with developmental language disorder (DLD) also exhibit different patterns of vocabulary development compared to children with TD, evidencing problems in many aspects of lexical acquisition (4). When DLD and bilingualism come together as in the case of bilingual children with DLD, diagnosis of lexical difficulties may be challenging. The purpose of the study is to describe the potential of a new word learning task, using a dynamic approach, to detect lexical-based language difficulties in monolingual and bilingual children with DLD.

Countless studies have reported low vocabulary scores in bilingual children compared to their monolingual peers, when assessed in one language, typically their second language (5–7). When both languages of the bilingual are taken into consideration, by calculating a total vocabulary score (i.e., adding words from both languages), bilinguals may outperform (6) or have equivalent results to monolinguals (8). Nevertheless, the “conceptual score,” which is obtained by counting the number of concepts known in at least one of the languages of the bilingual child, may still not be at the same level as monolingual peers depending on the child's language exposure patterns (9, 10). The main explanation for the poor performance of bilingual children on vocabulary tasks is the distributed characteristic of their knowledge (6). More precisely, bilingual children divide their time between two or more languages, and, depending on the context and exposure patterns, they do not develop the same vocabulary in each language: concepts can be known in one but not in the other language. Vocabulary development also depends on the age at which the second language is acquired. Two types of bilingualism are generally distinguished. Simultaneous bilinguals refer to children who learn both languages from birth or before 3 years, and consecutive bilinguals refer to children who learn their second language after the acquisition of their first language, often after 3 years (when they are entering school) (11). These two types of bilingualism generate different patterns of performance in terms of lexical knowledge. Simultaneous bilinguals show more balanced skills between their two languages (e.g. (12)), while consecutive bilinguals show more advanced skills in their first language than in their second (6). As a result when assessed in one language, consecutive bilinguals perform less well on English receptive vocabulary tasks (i.e., the non-dominant language of consecutive bilinguals) than simultaneous bilinguals (13). Vocabulary development not only depends on the age of second language acquisition but also on the amount of language exposure. Studies show that rate of vocabulary learning is proportional to the amount of exposure, which may change across time, according to the context of use (whether the language is spoken at home, school, or another location) (13–15). On expressive vocabulary tasks, it has been shown that at least 60% of exposure to the target language is necessary for bilinguals to perform as well as monolinguals (14). For simultaneous bilinguals, the language with greater exposure is associated with higher vocabulary scores than the language with less exposure (16). Finally, it's important to take into consideration the impact of Socio-Economic Status (SES) on vocabulary development. Even if Gathercole et al. (17) observed that home language exposure was a better predictor of vocabulary than SES, SES - at least as determined by parent educational levels – still had an impact on lexical performance, especially in older children. In a study looking at the separate and combined effects of SES and bilingualism on vocabulary, Meir and Armon-Lotem (18) showed that both SES and bilingualism contributed individually to vocabulary size without any interaction. In conclusion, according to MacLeod et al. (19), the vocabulary development of bilingual children is influenced by four main factors (as discussed above): the language of assessment, the age of second-language acquisition, the current language exposure and the sociolinguistic context.

DLD, formerly known as Specific Language Impairment, is a prevalent and persistent disorder that affects the expressive and receptive language skills of children. It develops during childhood and has an impact on everyday life, social interaction and academic progress (20). DLD affects several language domains including lexical skills, although vocabulary deficits are not universal in this disorder (see (21) for a review)**.** Children with DLD often have less vocabulary knowledge, both in breadth and depth (22). This means that they have fewer words in their lexical inventories (= breadth) and less semantic knowledge (= depth) about the individual words. At a qualitative level, vocabulary deficits impact rare or abstract words more than frequent and concrete ones (23), verbs more than nouns (24), and word forms more than meaning (25). Children with DLD also present “word finding difficulties”: they experience difficulty finding the target words (26). During development, they learn words more slowly than their peers (4).

The preceding discussion indicates that vocabulary weaknesses may be observed as a result of bilingualism and/or DLD. When the two come together as in the case of bilingual children with DLD, differential diagnosis may be particularly difficult. In order for bilingual children to be diagnosed as having DLD, they should have difficulties in both languages (27). Speech-Language Therapists (SLT) should, thus, assess children in all of their languages. However, assessment tools are not always available (there are few tests available in some languages) or appropriate for testing bilinguals (due to content and linguistic bias, or standardization only on monolinguals, e.g. (28)). These limitations make it difficult to evaluate the language skills of bilingual children as recommended. The main barriers reported by SLTs are access to adequate resources, including time, financial support, language support in the form of an interpreter, and training in bilingual assessment (29, 30). Due to these difficulties and the inappropriateness of assessing bilingual children's vocabulary, even in both languages, investigators have looked for alternative methods of assessing word knowledge such as measuring language learning (e.g., word learning) through dynamic assessment, which will be discussed below.

Despite its apparent complexity, learning new words appears to be easy for most children, but may be difficult for others, in particular those with DLD. Many theories and models have been developed to explain how children learn new words. Some models emphasize the different stages of word acquisition. Carey and Bartlett (31) for example, were the first authors to describe the notion of fast mapping, that is, the initial stage of word learning, in which an initial mapping between the phonological and semantic representation (word form and meaning) is made after minimal exposure. More recently, Leach and Samuel (32) distinguished lexical configuration which is “the set of factual knowledge associated with a word” (e.g., the word's form, meaning, and syntactic role) from the process of lexical engagement which is when “a lexical entry dynamically interacts with other lexical entries”. Furthermore, theoretical models highlight the strategies used by children for resolving referential ambiguity (i.e., determine which object a new word refers to). According to constraints and social-pragmatic models, children use attentional or perceptive (e.g., perceptual salience, temporal contiguity, and novelty), social-pragmatic (e.g., eye gaze, pointing, context/speaker intention) and linguistic (e.g., grammar, prosody) cues to map a new word to its referent and thus learn new words (33, 34). These models highlight the mechanisms, and the types of strategies and cues children use in word learning which will be useful for us when developing our word learning task.

As mentioned above, children with DLD experience difficulties in learning new words: they display significantly lower word learning performance than their TD age-matched peers, but they often have the same performance as their language-matched peers (see (4) for a meta-analysis). Several studies have thus found that children with DLD produce and comprehend fewer words than age-matched peers with TD in the same learning condition (35–38). More precisely, children with DLD require more exposure to acquire a new word (36, 39): they need up to twice the amount of exposure as children with TD to map word form and meaning (35). They notably require more support using cues (40) or more retrieval practice (i.e., more naming during the learning (41)) depending on the type of task. These deficits may be explained by difficulties in the initial encoding of the word form-meaning link (42). They may also be associated with difficulties in short-term memory and executive functions since the word learning performance of children with DLD has been found to be positively correlated with inhibition, verbal short-term memory and verbal working memory (37, 43), and it is well know that children with DLD have weaknesses in executive functions (44, 45)*.* Nevertheless, even if group differences are consistently observed between children with DLD and TD, there is high intra-group variability in DLD: some children perform similarly to their peers with TD, leading to moderate effect sizes and a lack of specificity that may impact the diagnostic accuracy of these tasks (4, 35).

As for multilingual children, studies reveal different results when comparing bilingual and monolingual performance on word-learning tasks. Some studies argue for a bilingual advantage due to the potential strengths of bilingual children in cognitive processes such as attention and memory (46, 47), and others for a monolingual advantage (48). Yet other studies observe similar accuracy between monolinguals and bilinguals, albeit different strategies (49). In Alt et al. (48), preschool bilingual children performed similarly to monolingual ones in receptive tasks (i.e., identifying names and linking names and referents) but were less accurate in production tasks (i.e., producing the learned words), whereas school-aged mono- and bilingual children performed similarly in all tasks. In a more recent study (50), the same authors employed a variety of word learning tasks to compare the performance of monolingual and bilingual children. They didn't find any differences between the two groups for most of the tasks, concluding that monolingual and bilingual children showed equivalent accuracy in word learning.

In sum, children with DLD experience difficulties in learning new words whereas bilingual children with TD seem to perform like their monolingual peers. Word learning could thus be a good indicator for diagnosing DLD in bilingual children. Nevertheless, there are many different types of word-learning tasks. They vary greatly from one study to another according to learning context, type of stimuli, task characteristics, and outcomes measures (51). As for the learning context, studies and meta-analyses show that shared storybook reading (SSBR) may support word learning (moderate to large effects) in children with TD (52–54). During SSBR, an adult reads a book to a child and the book contains new words to learn. Different factors influence the learning of words during SSBR: reading style (use of dialogic techniques, i.e., pointing, asking questions, etc.), number of exposures to new words (multiple exposures), and number of words tested (fewer words) leads to more word learning (55). In contrast, children's age, the person who reads the story, and the time between story and test have not been found to be moderating factors. In the case of DLD, Storkel et al. (56) showed that 34 children with DLD, aged 5–6, were able to learn new words after 36 exposures during interactive book reading regardless of the dose (i.e., the number of exposures to the target word during the session) and dose frequency (i.e., the number of sessions). The authors however reported that participants forgot newly learned words when the learning process was finished. Moreover, they found great variability between children in the number of words learned (from 0 to 14 out of 30 taught words).

Another way to test children's word-learning ability is *via* a dynamic assessment (DA) approach. DA represents an alternative (or supplementary) approach to traditional static language assessments, particularly suitable for bilingual children (57). It consists of assessing the child's learning potential during an interactive evaluation between the child and the examiner. It combines teaching, learning and evaluation and allows the examiner to focus on the “zone of proximal development” which corresponds to what the child can do with the help of an adult or a more-experienced peer (58). In this sense, DA is an evaluation which contains intervention: the examiner provides strategies or cues to the child to improve her/his performance. Graduated prompting, one method of DA, consists of giving prompts to the child during the evaluation until s/he succeeds at the task. Prompts are designed to become more and more supportive so as to provide an increasing amount of assistance (59). Another method of DA is the pre-test/teach/post-test approach in which the evaluation is composed of three steps: a pre-test in which static performance is measured, a teaching phase in which instructions are given to the child to succeed in the task, and a post-test in which the new performance following the intervention is measured. Finally, a central concept of DA is modifiability: Likert-scales are used to report on the child's behavior. At the end of the task, the examiner rates the child's attention, self-regulation, planning, and motivation (60, 61) and/or the examiner's effort and the child's responsiveness during the assessment (60, 62).

Numerous studies have evaluated word learning using a DA approach (40, 63–71). Kapantzoglou et al. (40) assessed twenty-eight Spanish-English bilingual children with low SES. They were 4–5 years of age and included 15 participants with TD and 13 with DLD. They performed a DA task, which consisted of learning three novel words paired with unfamiliar objects. At the pre-test, no child could name the novel objects. The learning context consisted in a scripted structured play activity during which a puppet received gifts for its birthday. Each word was presented nine times in each of three sessions. Post-tests consisted of asking the child to identify and name the objects. Modifiability scales were also rated by the examiner. Results showed that diagnostic classification was most accurate after the first post-test (i.e., after nine exposures): children with DLD performed significantly less well than those with TD on the identification of the novel words and on one of the modifiability scales. Even if their results were modest in terms of diagnostic accuracy, the authors concluded that their DA task may be a promising tool for the diagnosis of DLD in bilingual children. More recently, MacLeod and Glaspey (66) assessed twenty-six bilingual children with TD (aged 4–6) on a French adaptation of Kapantzoglou et al.'s task. They found an effect of word exposure with increasing performance in naming and identification of non-words across multiple trials. Moreover, scores were not correlated with age of exposure to the L2 (French).

Another type of word learning task in a DA format comes from Hasson et al. (65) who developed a DA battery referred to as the DAPPLE. It includes a subtest of vocabulary learning based on the work of Camilleri and Law (70). The authors tested twenty-six bilingual children (from diverse linguistic and cultural backgrounds): 12 with DLD and 13 with TD. The vocabulary task consisted of a pre-test assessing static receptive vocabulary which allowed the examiner to identify six words that the child didn't know. The teaching phase was a posting game: the child had to select three target picture cards (the unknown item among two known). Prompts were given if the child could not identify the correct picture. The post-test was an expressive task: children had to name the three pictures that s/he wanted to post. This procedure was repeated for three other unfamiliar objects. A second post-test was also conducted later: children had to name the six objects. Results showed that the DLD group needed more assistance in the teaching phase (i.e., they required more prompts to identify the target picture) than the control group. Moreover, this group performed less well than the control group in the second post-test which consisted in naming the objects, although there was no difference in the first post-test. According to the authors, this last finding was related to difficulty retaining or accessing the new words, rather than difficulty in distinguishing and encoding the phonetic information during learning.

As for French-speaking children, Matrat et al. (71) recently reported on three studies that used a word-learning task based on Kapantzoglou et al. (40) and Hasson et al. (65). In the first study, they found that bilinguals with TD performed less well than monolinguals on static measures of vocabulary knowledge but not on dynamic measures. In the second study, including only consecutive bilingual children, the DLD group performed less well than the TD group, but the difference between groups did not reach significance. In the third study, the dynamic measure was effective in distinguishing TD and DLD monolingual groups and did not discriminate between monolingual and bilingual TD groups on the phonological production task, which suggests that this word learning task could be an alternative approach to assess the lexical skills of bilingual children.

In the preceding literature review, we have discussed SSBR and dynamic approaches to word learning. Burton and Watkins (63) combined both approaches in a study of word learning in children with different SES backgrounds (low or high-risk background). The participants had to learn four novel words presented in a picture book narrated by the examiner. Each word was presented eight times in the story script and was pointed to one time at the first exposure. Questions to the child were embedded in the script, such as “They”re not very nice, are they?”. The child also had to complete a sentence for each target word during the story. During post-test, toys that corresponded to the novel items were given to the child who had to name them. Prompts were given if the child could not name the objects, including semantic cues (i.e., finding a picture of the item in the picture book and defining the object), phonemic cues (i.e., providing the first phoneme), and an indirect model (i.e., producing the word and repeating the elicitation question). Results indicated that the high-risk group performed less well than the low-risk group but that this difference was not significant. In general, word-learning scores were low for all children, although most children could name at least one of the four words without cues. Finally, semantic cues were less useful than the phonetic cues for naming a word.

More generally, most studies using DA to evaluate lexical development as well as other language domains such as syntax and narration show that children with DLD exhibit lower scores than their TD peers, and dynamic scores often give good diagnostic accuracy (see (72, 73) for systematic reviews). Nevertheless, DA is not used very often in clinical practice, principally due to lack of time, familiarity, and training by SLTs (29, 30). Yet, despite some methodological limitations, preliminary findings support the use of DA for the diagnosis of language disorders in multilingual children and indicate that DA is a time-efficient complementary method for language assessment (72). Thus, there is a need to develop a DA approach that is appropriate for clinical purposes. More precisely, tasks in French need to be developed and implemented in the clinical setting because bilinguals are the majority population among French-speaking Swiss SLTs (30).

To sum up, vocabulary weaknesses may be observed as a result of bilingualism and/or DLD. Diagnosis of DLD may therefore be particularly difficult for bilingual children, especially because of the lack of tools for SLTs to assess the lexical skills of bilingual children. Assessing word learning through DA seems to be an interesting alternative since children with DLD have difficulty learning words but bilingual children with TD do not. Moreover, dynamic word learning tasks have already shown good results with bilingual children with and without DLD. Nevertheless, there are few (if any) studies using DA in French and few which compare both monolingual or bilingual children, with TD or DLD. In the current study, we aim to improve the assessment of lexical skills in bilingual children by using a dynamic approach that targets word learning. Inspired by word learning and SSBR intervention studies, we have created a dynamic word learning task, using a SSBR approach. We aim to test its diagnostic accuracy in French-speaking monolingual and bilingual children. First, we will investigate which factors (e.g., developmental group, linguistic status, age, SES) impact the results of our task. Second, we will examine whether our dynamic measures are able to differentiate children with DLD from those with TD. For those measures that show significant differences between groups, we will calculate their diagnostic accuracy (sensitivity and specificity).

## 2. Materials and methods

This study was approved by the Ethics Committee of the Faculty of Psychology and Educational Sciences at the University of Geneva. Participation was voluntary and parents provided written informed consent for participation. The entire study was conducted in accordance with the Declaration of Helsinki.

### 2.1. Participants

Seventy monolingual and bilingual French-speaking children, aged 4;0 to 8;11, participated in the study. None of the children had hearing or visual impairments, genetic, neurological or other disorders (e.g., autism spectrum disorder or intellectual disability) based on a parental questionnaire. To rule out potential intellectual disability, we excluded participants (*n = 2*) who scored above −2 SD on a nonverbal reasoning (NVR) measure (Coloured Progressive Matrices (74)). The remaining participants (*n = 68*) were assigned to two groups: The Typically Developing (TD) and the Developmental Language Disorder (DLD) group. To be included in the DLD group, children had to be currently receiving speech-language therapy for an oral language disorder (different terminologies accepted: DLD, language disorder/problem/delay, speech delay, dysphasia, speaking difficulty) and to meet at least one of the following two criteria: 1) to have obtained a standard score below the cut-off (defined by the test) on a non-word repetition (NWR) task (see 2.3.2); 2) to have obtained a standard score below the cut-off on a sentence repetition (SR) task (see 2.3.2). These two criteria were chosen since impairment on repetition tasks constitutes a clinical marker of DLD for monolingual and bilingual children (75–77). To qualify for the TD group, children had to fulfil no more than one of the above two criteria. Eight participants who did not correspond to criteria for DLD and TD groups were removed: seven children, who were receiving speech therapy, didn't have a standard score below the threshold on NWR or SR tasks and one child, in the TD group, had two standard scores below the threshold on NWR and SR tasks. Our final sample thus consisted of 60 children: 43 children in the TD group and 17 children in the DLD one. Table 1 summarizes their demographic characteristics and indicates their mean scores on NVR tasks.

**Table 1:** Demographics characteristics and scores on NVR task of the sample, by group.

Group	N	Age range	Age	Gender		Linguistic status		SES	NVR: z scores
** **	** **	** **	Mean (SD)	F	M	ML	BL	Mean (SD)	Mean (SD)
**TD**	43	4;1–8;8	6;7 (1;5)	23	20	23	20	6.7 (5.7)	−0.05 (1.08)
**DLD**	17	4;4–8;2	6;7 (1;3)	5	12	9	8	2.8 (4.6)	−0.65 (0.82)

N, number; SD, standard deviation; TD, typical development group; DLD, developmental language disorder group; F, female; M, male; ML, monolinguals; BL, bilinguals; SES, education of parents (number of years of tertiary education); NVR, non-verbal reasoning.

A background questionnaire, based on the CECER-DLL (78) and the PABIQ (79), was developed to collect information on the participants' family and spoken languages. It comprised open and closed questions as well as Likert-scales about:

– Child demographics: birthdate, gender, country of birth, medical diagnosis and speech-language therapy;
– Language exposure and use of languages in the case of bilingual participants: age of exposure to French, amount of exposure before three years of age for each language, actual amount of exposure in each language, language dominance, level of comprehension and expression in the other language;
– Family demographics: age of parents, country of birth of parents, maternal languages, profession and education of parents, number of siblings.

Our sample included 32 monolingual and 28 bilingual children. Children were bilingual if their parents indicated that their child spoke another language at least 20% of the time in addition to French, following the results of Pearson et al. (15). Twenty-one (16 TD, 5 DLD) were simultaneous bilingual children and seven (4 TD, 3 DLD) were consecutive bilinguals. Two children spoke more than two languages. The languages spoken by the children, in addition to French, were: Arabic (*n = 10*), Spanish (*5*), Portuguese (*5*), English (*3*), Albanian (*2*), German (*1*), Armenian (*1*), Czech (*1*), Italian (*1*), and Kosovar (*1*). 51 parents (23 mothers, 28 fathers) spoke a language other than French as their first language.

The two developmental groups (TD, DLD) did not statistically differ in terms of age (W = 370, *p* = .95), gender (*χ*2 = 1.9527, df = 1, *p* = .16) and NVR (W = 483, *p* = .05), but they differed significantly on parental education level (W = 519, *p *= .006). The DLD group has a lower SES than the TD group, based on the number of years of parental education. This may be because children with DLD are more likely to come from families with low SES (see (80)).

### 2.2. Stimuli

#### 2.2.1. Non-words

Four non-words were created: /fuk/, /pitƐl/, /klan/, and /moze/. We used non-words to avoid possible partial knowledge of infrequent words by some children. They contained consonants acquired before 3 years (based on norms for Canadian French by MacLeod et al. (81)) and which are present in many languages (82). We also ensured that the phonotactic probability *(*i.e., the frequency with which sound segments occur in a language) and the neighborhood density (i.e., the number of words that differ from the target word by just one sound) of the non-words were low because studies indicate that word learning is better for words with low density and phonotactic probability (83–85).

#### 2.2.2. Novel objects

Four novel objects were created with common materials (such as a cork, a plastic disposable glass, wool, thumbtacks) to represent visual referents of the non-words. They had different sizes, shapes and colors. Each object had two semantic features: a category and a definition (see Table 2). We used concrete nouns because it's easier to map them to a visual referent and because nouns are less difficult to learn than verbs (86).

**Table 2:** Objects,semantic features and non-words associated.

Object	Category	Definition	Version 1	Version 2
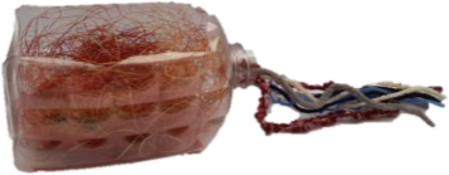	A car	It **flies**.	/pitƐl/	/fuk/
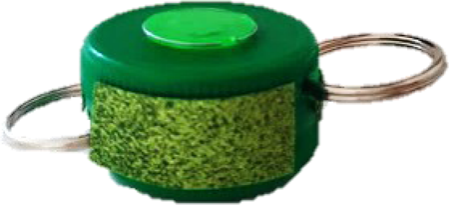	A machine	It finds **sweets**.	/fuk/	/pitƐl/
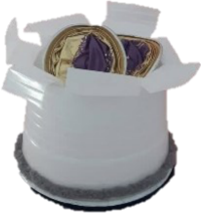	A hat	When worn, it helps the person to **jump**.	/moze/	/klan/
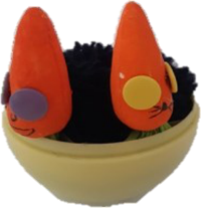	An animal	It likes **chocolate**.	/klan/	/moze/

In bold: the key word that the child had to recall.

#### 2.2.3. Story

A story (see Supplementary Appendix S1) was created around the novel objects which consisted of the adventures of a puppet who left in his car (object 1), taking a machine (object 2) and a hat (object 3). On the way, it met an animal (object 4). After the introduction of the novel objects, a disruptive event occurred: the puppet lost its bag. The following adventures consist in the puppet finding and recovering its belongings. The story ends with the puppet returning home.

During the story, each non-word was pronounced ten times and defined two times. In addition to the story script, two comments on each non-word were added to encourage examiner-child interaction: one request for repetition (e.g., *Look at his /moze/! Can you repeat /moze/?*) and one definition (e.g., *Would you like to jump with the /moze/ like Leo?*), while pointing to the target object (as in (53)). Also, on the penultimate occurrence of each non-word, the child was asked to complete a given sentence: the examiner read the beginning of the sentence and the child had to complete it with the target non-word. Whether or not the child succeeded, the correct form and the semantic features were produced by the examiner, and the child was asked to repeat the non-word (e.g., *It's a /moze/, a hat that allows you to jump! Can you repeat /moze/?*). In total, in the script and comments, each non-word was pronounced fifteen times by the examiner and repeated twice by the child; the definition was recalled four times and the category once by the examiner. In the storybook, each object appeared eight or nine times and was pointed to four times. The semantic feature corresponding to the definition was visible in the storybook on two occasions (e.g., picture of a sweet or chocolate). Two different non-word-object pairings were proposed to control for preference effects (e.g., /fuk/ refers to the machine in version one and to the car in version two). Thus, two versions of the story were constructed containing different object-name pairings: 37 children were given one version and 23, the other.

#### 2.2.4. Storybook

A storybook of twenty-two pictures was created to accompany the reading of the story (see Supplementary Appendix S2 for some examples). Each picture was made up of a background scene and elements (objects, characters) were added as focal points. The external images used were free of copyright (87). The size of the target objects was controlled so that the objects were always well visible even if the real proportions were not respected. The images created were printed in landscape format to form a book in which each image appeared alone (as in (88)).

### 2.3. Procedure

We recruited children with TD at schools and participants with DLD through contacts with SLTs, in France and Switzerland. Sessions took place at the school, home or speech therapist's office of the child and were conducted by the first author and two master students in speech-language therapy. Due to sanitary conditions, the examiners wore a mask during some sessions. Sessions were audio-recorded. All tasks were presented in the same order, in one or two sessions, for a total of forty minutes to one hour: 1) DA: teaching; 2) DA: immediate post-test; 3) Expressive vocabulary task; 4) DA: delayed post-test (about 10 min after the immediate post-test); 5) Non-word repetition (NWR) task; 6) Sentence repetition (SR) task; and 7) Non-verbal reasoning (NVR) task.

#### 2.3.1. DA: teaching phase

The teaching phase was preceded by the presentation of the novel objects. The examiner placed the objects in front of the child allowing him/her to look at them and manipulate them. After the children had touched and examined the objects, the examiner presented each object by pronouncing its name once and asking the child to repeat. The examiner then provided a second time the name and added the semantic features of the object. Following the presentation of the objects, the examiner explained to the child the course and purpose of the task, as recommended by Feuerstein (89) with the notion of intentionality (e.g., “*I'm going to read you a story where there are these funny objects. You are going to hear their names and you are going to try to learn them (…)”*). Finally, the examiner read the story, while interacting with the child, during approximately 10 min.

#### 2.3.2. DA: testing phase

After the learning phase, immediate and delayed post-tests assessed recall of the phonological form of the non-word (i.e., the child had to name the non-words) and the semantic features (i.e., the child had to describe the characteristics of the novel objects). The test procedures, including prompts, were presented in this order:

1. Phonological recall and prompts: each object was presented to the child who had to recall its name (i.e., the non-word): “*What is it called?”*. For those names that were not recalled or mispronounced, prompts were then given, as shown in Table 3.
2. Semantic recall and prompts: the child was asked to recall the semantic features of each object by answering the questions: “*What is it? What did you learn about this?*”. For those semantic features that were not recalled, specific prompts were then given (see Table 4).

**Table 3:** Phonological recall: prompts and scores.

Level	Prompt	Score/item
**0. Phonological recall**	The examiner asks: “*What is it called?*”	6
**1. Second trial**	The child tries again to recall the nonword.	5
**2. Phonemic prompt 1**	The examiner provides the first phoneme of the word (or the cluster /kl /for /klan/).	4
**3. Phonemic prompt 2**	The examiner provides the first two phonemes of the word (or /kla/ for /klan/).	3
**4. Recognition**	The child has to recognize the target word among two phonological distractors (e.g.: “*Is it /fum/, /fup/, or/fuk/?”*).	2
**5. Repetition**	The child has to repeat the correct form.	1 if correct 0 if incorrect

**Table 4:** Semantic recall: prompts and scores.

Level	Prompt	Score/item
**0. Semantic recall**	The examiner asks: “*What is it?*”	5
**1. Precision**	The examiner asks the child to provide more details: “*Can you say a little more?*”	4
**2. Storybook**	The examiner shows the child a picture in the book where the target object is present alone and asks “*Can you say a little more?*”.	3
**3. Precise question and completion**	The examiner asks a more precise question and begins a sentence that the child must complete: – For the category: “*Without telling me the name, can you tell what it is? It's a kind of…*” – For the definition: “*What does it do? It's used for…*” or “*It likes what? It likes…*"	2
**4. Recognition**	The child has to recognize the target word along with two semantic distractors (e.g.: “*Is it a kind of animal, flower, or ball?*”).	1 if correct 0 if incorrect

After each correct response or if the child was not able to name the object or recall its semantic features after the prompting, the examiner recalled the correct non-word and semantic features. The same procedure was repeated for the delayed post-tests.

#### 2.3.3. Expressive vocabulary task

An expressive vocabulary task was administered to assess children's static lexical knowledge. Because of the wide age range of children tested, it was necessary to administer two different vocabulary tests. Children in kindergarten were tested with the short version of the “naming” subtest of the French language evaluation battery EVALO (90) which includes 40 images (32 nouns and 8 verbs). Older children were tested with the “naming” subtest (short version) of the French Battery EVALEO (91) which includes 31 images (15 nouns, 4 adjectives and 12 verbs). The procedure was the same for both tasks: the child had to name the picture (or the action represented). We calculated a percentage based on the number of correct items divided by the number of items in the task in order to perform analyses on the whole group.

#### 2.3.4. Non-word/sentence repetition and non-verbal reasoning

Three tasks were performed as part of the inclusion criteria protocol, as mentioned previously. The NWR tasks were taken from the EVALO or EVALEO batteries, depending on the child's age. For each task, the child had to repeat 24 non-words of increasing difficulty (i.e., with an increasing number of syllables). Each non-word and syllable were scored correct (1) or incorrect (0). The SR task was taken from the French version of the CELF-5 battery (92). The children had to repeat sentences of increasing length until they reached the stopping criterion (i.e., four failed sentences in a row). Each sentence was scored on a scale from 0 to 3 according to the number of errors made (i.e., any morpheme changed, added, substituted, or omitted). We also used the Coloured Progressive Matrices (74) which consisted of 36 sheets of matrices of different patterns and colors. The participant had to correctly select the missing part. For these tasks, standard scores were calculated according to the standardization protocol in the manual. To control for inclusion criteria, the cut-off scores were selected according to the criteria of the manual itself (a standard score of 1 for the NWR subtest of EVALEO, and a standard score below 7 for the SR subtest of the CELF-5) or defined at – 2 SD (for subtests of EVALO and for the Matrices).

### 2.4. DA: scoring

During the DA, different measures were collected, which are detailed in Table 5.

**Table 5:** DA: measures and scoring.

Measures	Timing	Description	Score/item	Total score
**Repetition**	Before teaching	The child has to repeat the name of the object during the first presentation.	PCC	0–4
**Completion**	During teaching	The child has to complete the sentence in the story by providing the name of the object.	PCC	0–4
**Phonological prompts**	Post-tests: immediate and delayed	The child has to say the name of the non-word. For non-recalled non-words, the examiner provides prompts.	0–6	0–24
**Semantic prompts**	Post-tests: immediate and delayed	The child has to recall the semantic features of the object: the category and a definition. For non-recalled semantic features, the examiner provides prompts.	0–10	0–40

PCC, percentage of consonants correct for each non-word (number of correctly produced consonants divided by the total number of consonants in the target non-word and possible consonant additions).

### 2.5. Data analysis

Data were formatted using Excel software (93) and statistical analyses were performed with R software (94), using packages lme4 and cutpointr. We performed several preliminary analyses for group comparisons. We ran generalized linear mixed models (using two different dependent variables: Phonological and Semantic Prompts) to investigate which factors (e.g., linguistic status, age, SES) influenced the results of our task. The fixed predictor variables were:

– Task characteristic: version (non-word/object pairing)
– Participant characteristics: age, gender
– SES: parental education (number of years of tertiary education, following secondary school)
– Time (immediate or delayed post-test)
– Developmental group (TD or DLD)
– Linguistic status (monolingual or bilingual)

Random effects were participants and items. Finally, we calculated sensitivity and specificity scores of DA measures to examine whether our dynamic measures were able to differentiate children with DLD from children with TD and to what degree.

## 3. Results

### 3.1. Preliminary analyses

Table 6 shows the results for TD and DLD groups on static and dynamic tasks. At a qualitative level, we observe that phonological prompt scores give lower scores than semantic prompt scores. Then, we performed preliminary analyses on the results of the static measures of DA (repetition, completion) and the static expressive vocabulary task. On the repetition measure before the reading of the story, TD and DLD groups did not differ in their ability to repeat the non-words before they were learned (W = 358, *p *= .99). For the static scores during DA, when the child had to complete a sentence with the target non-word, significant differences were found between TD and DLD groups (W = 505, *p* = .02), with the TD group performing better than the DLD one. Turning to static vocabulary knowledge, Wilcoxon tests showed that the TD group performed significantly better than the DLD group (W = 568.5, *p* < .001) and monolingual children performed significantly better than bilingual children (W = 656.5, *p *< .01). More precisely, pairwise comparisons on subgroups showed that the monolingual TD group performed better than all other groups, including the bilingual TD group (W = 370, *p* = .004 with TD-BL; W = 170, *p* = .036 with DLD-ML; W = 176, *p* < .001 with DLD-BL, see Table 7).

**Table 6:** Means (SDs) on each measure according to developmental group.

Task	Measures	TD	DLD
**Dynamic Assessment**	Repetition (/4)	3.9 (0.3)	3.9 (0.3)
	Completion (/4)	1.7 (1.3)	0.9 (0.8)
	Immediate Phonological Prompts (/24)	14.8 (4.7)	9.4 (4.5)
	Delayed Phonological Prompts (/24)	11.6 (5.1)	8.0 (3.3)
	Immediate Semantic Prompts (/40)	29.5 (6.6)	26.0 (6.5)
	Delayed Semantic Prompts (/40)	35.2 (4.7)	31.4 (6.6)
**Static expressive vocabulary**	Percent correct	67.2 (16.6)	49.4 (17.8)

TD, typical development group; DLD, developmental language disorder group.

**Table 7:** Means (SDs) on expressive vocabulary and semantic measures.

Task	Measure	Score	TD		DLD	
** **	** **	** **	Monolingual	Bilingual	Monolingual	Bilingual
**Dynamic Assessment: Semantic Prompts**	**Immediate post-test**	Category (/20)	10.2 (4.9)	12.6 (4.1)	10.8 (4.7)	9.8 (3.2)
		Definition (/20)	17.8 (2.8)	18.7 (2.8)	16.0 (3.4)	15.4 (4.6)
		Total (/40)	28.0 (6.9)	31.3 (6.0)	26.8 (6.3)	25.1 (7.1)
	**Delayed post-test**	Category (/20)	14.3 (4.8)	17.7 (3.2)	14.9 (3.1)	12.6 (4.3)
		Definition (/20)	19.4 (1.4)	19.4 (1.2)	17.8 (2.7)	17.4 (4.2)
		Total (/40)	33.7 (4.9)	37.0 (3.9)	32.7 (5.3)	30.0 (8.0)
**Static expressive vocabulary**	Percent correct		75.3 (10.1)	58.0 (18.1)	51.9 (21.8)	46.5 (12.9)

TD, typical development group; DLD, developmental language disorder group.

### 3.2. Factors influencing the dynamic measures

We investigated which factors influenced the results of our dynamic task by conducting generalized linear mixed models with phonological and semantic prompts as dependent variables. We first fitted a model without including interaction effects between independent variables and we then fitted models including two interaction effects: the interaction between developmental group and time (to know if the possible effect of group was present across the two post-tests) and between developmental group and linguistic status (to know if the possible effect of group was present regardless of the linguistic status of the children). The final optimal model (presented here) was the one with the best model fit and parsimony according to Akaike information criterion (AIC) and the Bayesian information criterion (BIC).

For phonological prompts, the model containing the interaction between time and developmental group led to the best-fitting model. Results are presented in Table 8. We found an effect of version, age, time, and developmental group. We also found marginal effects of gender, linguistic status and of the interaction between time and developmental group. The main effects of time and developmental group were also found on the model without interaction (see Supplementary Appendix S3). For semantic prompts, the model with both interactions was superior. The results of this model are presented in Table 9. We found an effect of version, age, gender, time, linguistic status and of the interaction between developmental group and linguistic status. The interaction between time and developmental group was marginally significant. Note that the main effect of linguistic status was not found in the model without interaction (see Supplementary Appendix S3).

**Table 8:** Results on generalized linear mixed model for phonological prompts.

** **	Reference	Estimate	Std. error	Z value	Pr (>|z|)
**(Intercept)**		0.74806	0.20902	3.579	0.000345***
**Version**	2	−0.47057	0.19658	−2.394	0.016674*
**Age (month)**		0.52155	0.10577	4.931	8.18e–07***
**Gender**	Boys	0.38238	0.20901	1.829	0.067331
**Education of parent**		0.12499	0.10893	1.147	0.251186
**Time**	Delayed	−0.60202	0.09702	−6.205	5.47e–10***
**Developmental group (DG)**	DLD	−1.12506	0.23966	−4.694	2.67e–06***
**Linguistic status (LS)**	Bilingual	−0.32203	0.18335	−1.756	0.079033
**Time x DG**	Delayed/DLD	0.33264	0.18113	1.836	0.066291

p = <.10 (*); < .05 *; < .01 **; < .001 ***.

**Table 9:** Results on generalized linear mixed model for semantic prompts.

** **	Reference	Estimate	Std. error	Z value	Pr (>|z|)
**(Intercept)**		0.88444	0.31748	2.786	0.00534**
**Version**	2	−0.4796	0.2107	−2.276	0.02285*
**Age (month)**		0.5554	0.1113	4.990	6.03e–07***
**Gender**	Boys	0.5341	0.2219	2.407	0.01609*
**Education of parent**		0.1756	0.1203	1.459	0.14447
**Time**	Delayed	1.0912	0.1003	10.885	< 2e–16***
**Developmental group (DG)**	DLD	−0.07047	0.31367	−0.225	0.82224
**Linguistic status (LS)**	Bilingual	0.6726	0.2355	2.856	0.00429**
**Time x DG**	Delayed/DLD	−0.2998	0.1664	−1.802	0.07161
**DG x LS**	DLD/bilingual	−1.1043	0.4217	−2.619	0.00882**

Education of Parent, number of years of tertiary education; DLD, developmental language disorder group;.

p = <.10 (*); < .05 *; < .01 **; < .001 ***.

Focusing on semantic scores, we ran the same models with the semantic sub-scores (category and definition) to determine if one of the two semantic features better explained the results. The best-fitting model was the one with both interactions for category as dependent variable and the model without any interaction effects for definition as dependent variable (see Supplementary Appendix S3 for detailed results). The effects of age and time were found for the two features, but the effects of version, gender and the interaction between developmental group and linguistic status were found only for category. For definition, a main effect of developmental group appeared. Moreover, for category, the interaction between time and developmental group was significant.

To sum up the findings of the statistical models, for all dynamic measures (phonological and semantic), we found an effect of age. As age increased, children obtained higher dynamic scores. Moreover, we found an effect of time for all dependent variables, but with different patterns: immediate post-test was associated with better results for the phonological prompts but with lower results for the semantic prompts (for both category and definition scores) than delayed post-test. The effect of developmental group (TD, DLD) was found for phonological prompts and the definition score: children with TD had higher scores than children with DLD. For phonological and semantic prompts, a marginal interaction between time and developmental group emerged. For phonological prompts, pairwise comparisons showed that there was a difference between TD and DLD groups at both post-tests (W = 8192, *p* < .001 for immediate post-test, W = 7746, *p* < .001 for delayed post-test), but the difference between immediate and delayed post-test was found only for the TD group and not for the DLD group (W = 17,648, *p* < .001 for TD, W = 2416, *p* = .6 for DLD). For semantic prompts, this interaction was significant only for the category score. Pairwise comparisons showed that children with TD did not perform better than children with DLD at immediate post-test (W = 6298, *p* = .335), but marginally better at delayed post-test (W = 6592, *p* = .07), and the improvement of category scores between immediate and delayed post-test was found for both developmental groups (W = 9708, *p* < .001 for TD; W = 1771, *p* = .01 for DLD). For semantic prompts, the interaction between developmental group and linguistic status was found only for the category score. Pairwise comparisons showed that the TD bilingual group performed better than TD monolingual and DLD bilingual groups for the recall of the category (W = 17,697, *p* = .003 with TD-ML; W = 3852, *p* = .007 with DLD-BL, see Table 7). Finally, we found an effect of version: version one led to better scores than version two for phonological prompts and category scores. An effect of gender was also found for semantic prompts (and a marginal effect was found for phonological prompts): boys performed better than girls when recalling categories.

### 3.3. Discriminant validity

Although our dynamic measure of phonological prompts was able to distinguish between children with and without DLD, this does not allow us to conclude that our task is a useful diagnostic tool. Thus, we also determined its diagnostic accuracy by calculating sensitivity and specificity indices (95, 96). Sensitivity refers to the degree to which children who were previously classified as DLD will be categorized as language impaired by the test. Specificity is the degree to which children who were classified as TD will be identified as unaffected by the test (e.g. (96)). According to Plante & Vance (97), sensitivity and specificity values are considered good when they are above 90%, fair when they are between 80%–89%, and unacceptable when they are below 80%. In order to determine the sensitivity and specificity of a measure, it is necessary to determine the cut-off score which distinguishes between participants with and without a disorder on that measure. In a clinical setting, SLTs often use arbitrary cutoff scores (e.g., 1, 1.5 or 2 SDs under the mean, see (98)) to determine the presence or absence of a disorder. However, this practice does not always lead to accurate diagnoses, as children with DLD often do not score below these cutoffs (99). The cutoff scores are in fact different from one test to another (97). Different methods have been developed to determine the optimal cut-off values for quantitative diagnostic tests (see (100)). We chose to use the method based on the calculation of the Youden index that maximizes the sum of sensitivity and specificity.

**Figure 1:**
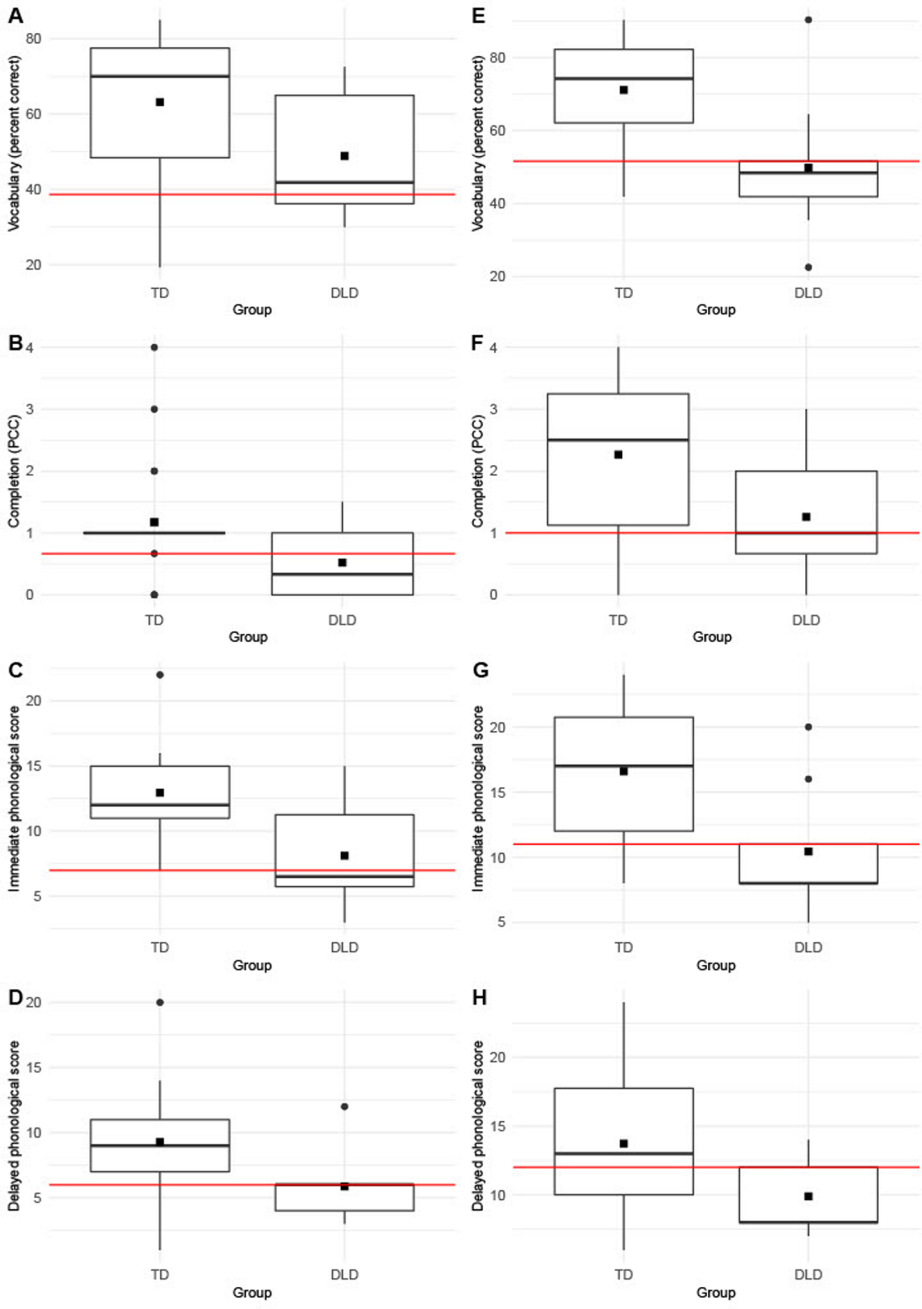
Boxplots of the results on each measure according to age group. (**A–D**): Results for the group of children between 4 and 6 years; (**E–H**): Results for the group of children between 7 and 8 years; Red line represents the optimal cut-off. The square represents the mean.

We examined the diagnostic accuracy of our dynamic measures as compared to that of the static measures. For DA, we focus only on the phonological production measures since they were the only ones that revealed significant differences between TD and DLD groups. For static measures, we focus on the scores obtained on the expressive vocabulary task and on the completion task conducted during the SSBR. Because we found an effect of age on our dynamic measures, we separated our sample into two groups to perform these analyses: children aged 4–6 years (21 TD, 8 DLD) and children aged 7–8 years (22 TD, 9 DLD) (see Figure 1 to view the results for these two groups). Table 10 shows the optimal cut-offs according to the Youden index and the percentage of sensitivity and specificity for each measure.

**Table 10:** Diagnostic accuracy for each measure according to age group.

Age	Indices	Vocabulary	Completion	Immediate Phonological Prompts	Delayed Phonological Prompts	Total Phonological Prompts
**4-6**	Optimal cut-off	38.7	0.67	7	6	18
	Sensitivity	50%	62.5%	62.5%	87.5%	87.5%
	Specificity	90.5%	81.0%	95.2%	90.5%	76.2%
	Youden Index	0.4048	0.4345	0.5774	0.7798	0.6369
**7-8**	Optimal cut-off	51.61	1	11	12	23
	Sensitivity	77.8%	66.7%	77.8%	88.9%	77.8%
	Specificity	90.9%	72.7%	77.3%	54.6%	72.7%
	Youden Index	0.6869	0.3939	0.5505	0.4343	0.5051

For children between 4 and 6 years, all dynamic measures showed a better Youden index than those on the vocabulary task and the completion measure. The maximum Youden index was found for phonological prompts at delayed post-test, with sensitivity and specificity scores above 80%. More precisely, this measure led to fair sensitivity and good specificity (according to Plante & Vance, 1994). The static measures of the vocabulary task and the completion task showed unacceptable sensitivity.

In contrast, for older children, aged between 7 and 8 years old, the static expressive vocabulary task gave the best Youden index and led to good specificity and almost fair sensitivity. The immediate post-test of phonological prompts then showed the next highest percentages of accuracy, with the two indices approaching 80%, a better result than the static completion measure.

### 3.4. Qualitative analysis of the use of prompts

Table 11 shows the percentages of successful responses corresponding to each level of prompt, which allows us to analyze the usefulness of the individual prompts at a qualitative level. For Phonological Prompt scores, the second trial of phonological recall (level 1) and the phonemic prompts (levels 2 and 3) rarely led to successful recall. The most helpful cue in both situations was recognition, that is, providing the child with three possible non-words of which one was the target form (level 4). Free recall led to 33.75% in the immediate post-test and 16.67% in the final post-test, a strong decrease in recall without cues being evident from immediate to delayed post-test. For Semantic Prompts scores, free recall (level 0) yielded the highest percentages of success. There was an effect of post-test: scores improved from immediate to delayed post-test. In addition, there was an effect of semantic feature: definitions were easier for children than categories. For recalling the category, recognition (level 4) was very useful especially on the immediate post-test, even though many errors were found. The use of a precise question with a sentence to complete (level 3: “*Without telling me the name, can you tell what it is? It's a kind of…*”) also helped the children. For providing a definition, returning to a picture from the storybook (level 2) on the immediate post-test allowed most children who had not recalled the definition in free recall to recover the meaning of the object.

**Table 11:** Percentage of successful responses for each level of prompt.

Level	Phonological Prompts		Level	Semantic Prompts			
** **	Immediate	Delayed		Immediate		Delayed	
** **				*Category*	*Definition*	*Category*	*Definition*
**0**	33.75	16.67	**0**	37.08	74.17	64.17	87.08
**1**	0.83	1.67	**1**	6.25	4.58	3.33	5.42
**2**	2.08	3.33	**2**	1.67	12.50	0.83	2.92
**3**	5.0	7.50	**3**	16.67	2.92	16.25	1.67
**4**	44.58	55.42	**4**	26.67	5.0	12.50	2.50
**5**	12.50	12.92	**Incorrect**	11.67	0.83	2.92	0.42
**Incorrect**	1.25	2.50	** **				

## 4. Discussion

The aim of this study was to improve the assessment of lexical skills in bilingual children by using a dynamic approach that targets word learning. Inspired by word learning and SSBR intervention studies, we created a dynamic word learning task, using a SSBR approach and we tested its diagnostic accuracy in French-speaking, monolingual and bilingual, children with and without DLD. Our task involved the recall of phonological and semantic information of novel objects learned during a SSBR, which we tested immediately after the SSBR and after a delay of 10 min. We provided phonological and semantic prompts to help children recall the information when they were unable to do so. We conducted generalized linear mixed models to examine the factors that influence dynamic test performance and determined the diagnostic accuracy of our task by calculating its sensitivity and specificity. Our statistical models indicated that age, time and developmental group (TD, DLD) influenced phonological prompts whereas age, time and interaction between developmental group and linguistic status (monolingual, bilingual) influenced semantic prompts. We found that diagnostic accuracy was acceptable for the delayed phonological prompts post-test in the younger group of children but that it was less good than a static measure for the older group of children. We now discuss the findings in more detail.

### 4.1. Word learning skills

The first aim of the study was to investigate which factors (e.g., linguistic status, age, SES) impact the results of our dynamic task. We found a main effect of age for all measures suggesting that word learning skills increase with age. Few studies have investigated differences between younger and older children on word learning tasks (4). Age-related factors, such as cognitive maturation, language exposure, and school experience may play a facilitating role in word learning in school-aged children (see (4)). Indeed, the development of attention and other cognitive skills during childhood enables the child to better select and process contextually relevant linguistic information (101), and thus to perform better on a word learning task.

The outcome measure of our task targeted two aspects of word learning: the recall of the phonological form of the word and the recall of the semantic features of the object. We found that children had more difficulty recalling the form of the word than the meaning. Other studies have found that learning the word's form is more difficult than learning the word's meaning, and that this is especially the case for children with DLD (36, 42, 102). Moreover, different variables affected the results of these two measures.

For the phonological score, we found an effect of developmental group: children with TD performed better than their peers with DLD on this measure, with no significant effect of linguistic status. McGregor and colleagues (42) have explained that children with DLD are less accurate in linking words to their referents than their typically-developing peers because of weaknesses in initial encoding. We also observed this difficulty of initial encoding, especially since our task involved fast mapping with exposure to the new non-words during a short period. Moreover, the results decreased between immediate and delayed post-test, for both groups, however only significantly so for children with TD. This can be explained by the fact that children with DLD already have very low initial results. In general, most children displayed difficulty in recalling the non-words learned in the story. They often needed to be provided with a choice of phonological forms (i.e., the target form and phonologically close distractors) to find the target word. Helping the children by giving them the first phoneme(s) (i.e., phonemic prompts) of the word was not sufficiently effective. Thus, the initially encoded phonological representation was not strong enough to enable children to retrieve the correct word form even when phonological cues were provided. Moreover, this weak encoding did not allow retention and/or access to the word after 10 min. We might have expected higher scores on the delayed post-test since the immediate post-test provided another opportunity for word learning. Regardless of the child's response at immediate post-test, the examiner provided the correct form of the word. We could therefore consider this test as a final learning trial, just as completion was during reading. Both of these measures (i.e., completion and immediate post-test) could be thought of as retrieval trials, and various studies have shown that retrieval during learning leads to better word retention (see (38) for multiple studies). In our study however, hearing the phonological form of the non-word at immediate post-test did not allow the children to perform better on the delayed post-test, probably because a single retrieval event was not sufficient to aid phonological encoding. Our word learning study thus differs from other retrieval-based word learning studies which contain greater numbers of retrieval trials.

For the semantic scores, we found a different pattern: scores increased between immediate and delayed post-test for both the recall of categories and definitions. Here, the retrieval attempt and the exposition to the correct answer allowed children to better encode the semantic features and thus to improve their performance. The other effects found for the semantic score (i.e., the effect of version, gender, and the interaction between developmental group and linguistic status) were only found in the model based on category information. Interestingly, an effect of developmental group was found for the definition score. Although the results were high in both groups, the group with TD performed better than the group with DLD when recalling definitions. For category information, a marginally significant difference between the two groups was found at delayed post-test. The different effects of time and developmental group observed between the recall of categories and definitions may be related to the quantity and type of exposure to each feature in the word-learning task: the category was said only one time whereas the definition was said four times and was visible in the storybook (e.g., the pictures show the car flying, the animal eating chocolate). Because of this exposure condition, the definition was learned more easily, especially for the TD group who displayed better scores than the DLD group on both post-tests, despite the ceiling effects. Moreover, children had more difficulties to recall the category at immediate post-test, but once they were given the category *via* the prompts on the first post-test, they were better able to recall the category on the delayed post-test. Consequently, the results on category scores increase, especially for the TD group, leading to a marginal difference at the delayed post-test between the TD and DLD groups. Finally, for the category sub-score, we also found a significant effect of the interaction between developmental group and linguistic status: the bilingual TD group performed better than two other groups. It is unusual for bilinguals to perform better than monolinguals on lexical semantic tasks (22, 103). Nevertheless, Rosqvist and colleagues (103) showed that when explanatory factors such as SES or school experience were entered into their statistical model, the negative effect of bilingualism was reduced. In addition, they found that language skills assessed by static tests explained the scores on the definition task better than any of the other factors. We assume that the advantage displayed by the bilingual children reported in our study was due to the specific characteristics of our population: our bilingual participants were dominant in French and had many years of exposure to French, especially at school. Crucially, bilingual children did not perform less well in either phonological or semantic recall. Our dynamic measures thus did not penalize bilinguals with respect to their semantic recall.

Finally, we found an effect of version for phonological prompts and category scores that is difficult to explain because, even if some items were better recalled than others (e.g., the non-word /pitƐl/ was better recalled than /klan/ and/moze/, and the semantic category “car” was better recalled than the “machine” and “hat”), the matching between these items in one version or the other does not allow to conclude why one version was more successful. We also found an effect of gender for category scores and a marginal one for phonological scores. It is unexpected that boys perform better than girls on the recall of definitions. Other studies have instead shown an advantage for girls in word learning tasks (e.g., (104)). We assume that the advantage displayed by the boys reported in our study was due to the specific characteristics of our population. Finally, no effect of SES was found for all measures suggesting that SES did not influence our task.

### 4.2. Diagnostic power of the task

The second aim of the study was to determine the diagnostic accuracy of our test. We found that results on the phonological prompts measure (i.e., children had to recall the name of the objects and received prompts when they had difficulty) was able to distinguish children with TD and DLD. Children with TD performed better than children with DLD on these measures, at immediate and delayed post-test. More precisely, these dynamic measures had better sensitivity and specificity scores for young children between 4 and 6 years than the static measure of expressive vocabulary and the completion task conducted during the SSBR. The delayed post-test revealed diagnostic accuracy scores of fair sensitivity and good specificity whereas the immediate post-test displayed poorer sensitivity. This result is in line with Hasson et al. (65) who found a group difference only at the second naming post-test. We aimed for this effect to be independent of bilingualism and SES and, indeed, we found that the results of the phonological prompt measures were not affected by bilingualism or SES scores in our statistical models.

Nevertheless, for children aged 7 to 8 years, dynamic measures revealed diagnostic accuracy indices below 80%, not allowing us to correctly classify children with and without DLD. Thus, our task seems more appropriate for diagnosing young children. In their meta-analysis, Kan & Windsor (4) also showed that the difference between TD and DLD groups on word learning tasks is larger for preschoolers than for school-aged children.

The completion task at the end of the SSBR also differentiated children with TD and DLD, but showed unacceptable levels of sensitivity (and specificity for the older group). The completion measure therefore seems less sensitive than dynamic measures using prompts. Phonemic prompts (i.e., providing the first phoneme(s) of the word), while not always helping the child recover the word, contributed to a hierarchical score which was better able to classify children at the individual level than the static completion task. Indeed, the total score for completion was out of 4 while the one for prompts was out of 24. The rank of the scores being wider for the latter measure allowed for a more precise cut-off that better distinguished children with or without DLD.

We have seen that the sensitivity and specificity scores were not always above 80%, although for most of the dynamic measures, they were close. This suggests that some children with DLD perform like children with TD and that some children with TD do not have completely normal scores. These results have already been found in other word learning studies where group differences have been accompanied by large variability (35, 105)*.* This is why, in practice, one does not use a single index to define a diagnosis. Here, we only focus on lexical skills and more precisely on word learning performance, but not all children with DLD have difficulties in learning new words (4). The lower sensitivity found on some measures may therefore be explained by the fact that some children with DLD actually have less difficulty in this area. In our sample, we selected children with DLD without looking at the language profiles of the children. However, it is known that children with DLD present lexical disorders less often than phonological or morpho-syntactic disorders (e.g. (106)). This could explain why the diagnostic power of our task was not always good. It would therefore have been better to recruit only children with lexical disorders. Nevertheless, it would have been extremely difficult to do so since lexical disorders are precisely what are misdiagnosed in the bilingual population, when using static tests.

We expected that the static expressive vocabulary task would not show good diagnostic accuracy since this kind of task is generally not considered appropriate for bilingual children (5, 107). Using this measure, we found a group difference according to developmental group (with the TD group outperforming the DLD group) as well as a difference in linguistic status (with the monolinguals having better scores than the bilinguals). However, this difference in linguistic status was only found in children aged 4 to 6 years, when the analyses were performed on the subgroups. Concerning diagnostic accuracy of the static task, we found different results depending on the age of the children. For children aged 4–6 years, the task showed poor sensitivity: children with disorders were not diagnosed as such. For children aged 7–8 years, sensitivity was better and approached an acceptable threshold. Thus, in our sample, only the younger bilingual group seemed to be penalized on the naming task. More precisely, young bilingual children with TD performed similarly to young monolingual children with DLD in this task; the overlap between the results of these 2 groups led to an incorrect classification. On the other hand, older bilingual children with TD performed better than monolingual and bilingual children with DLD. In our sample, many of the bilinguals were exposed to French at birth and were dominant in French; we thus can assume that older TD bilingual children perform better because they have been exposed to French for a longer time.

### 4.3. Limitations

Concerning the limitations of our study, we have identified some design issues. First, we did not control whether the non-words used were existing words in the various L1s of the bilingual children. This would have been difficult to do in our study because we did not know the other languages spoken by the children before the testing began. Second, recall of semantic features was not sensitive enough to discriminate between children with and without DLD, consistent with the frequent finding that recall of semantic features is easier than recall of phonological information (36, 42, 102). The higher scores for semantic features could also be explained by the fact that, in our semantic task, the child could see the object and thus easily describe its appearance or say what it was. If we had asked the child to define the word, it would have been a more difficult task. Finally, phonemic prompts (i.e., saying the first phoneme(s) of the non-word) were not very useful: children principally recalled non-words without cues or during recognition. This is consistent with the results of a study by Gray (37) who showed that giving phonological cues during learning does not help children with TD to retrieve the word.

Finally, we used NWR and SR tasks to determine *a priori* whether children were in the TD or DLD group. These tasks were chosen because different studies show that they allow a correct diagnosis of DLD (75–77). This a-priori classification therefore seems appropriate because we used multiple sources of evidence to confirm diagnosis, especially for bilingual children (108), but it is possible that children were still not correctly classified. Indeed, the tasks used may not be the best possible. For example, the NWR task we used proposes more and more complex words and is based on the phonology of French whereas a language-independent task, such as that of Dos Santos & Ferré (109), may give more sensitive results. Indeed, a meta-analysis showed that the use of quasi-universal tasks results in higher effect sizes than language-specific tasks, which depend more on language experience (77). Unfortunately, these tasks are not available and standardized in French. More generally, since there is no gold standard for diagnosing DLD, tests used as a reference vary between studies (72) In short, we cannot be sure that our pre-inclusion of children in the DLD group was entirely correct. This leads to another limitation of the study regarding the number of children with DLD in our sample. Our final sample was small although we initially tested 24 children receiving speech-language therapy; however, seven who performed within the norms on reference (i.e., repetition) tasks had to be excluded.

## 5. Conclusion

Our findings suggest that a dynamic word learning task using SSBR is a promising approach for diagnosing lexical difficulties in young French-speaking monolingual and bilingual children. Children with DLD experienced more difficulties recalling the phonological form of the word than their peers without DLD. Following these promising results, we have created a new SSBR task by better controlling semantic features and choosing more replicable objects (110). We have also developed a word learning task with retrieval trials during learning based on of the work of Leonard and colleagues (38). Their experiments have shown that repeated spaced retrieval results in greater recall of word form and meaning. Thus, we hope that with further refinement of our dynamic word-learning approach, we will obtain better specificity and sensitivity scores and that our task can be eventually employed in the clinical setting for the diagnosis of DLD in French-speaking monolingual and bilingual children.

## Data Availability

The raw data supporting the conclusions of this article will be made available by the authors, without undue reservation.
