# Erratum: A new dynamic word learning task to diagnose language disorder in French-speaking monolingual and bilingual children

**DOI:** 10.3389/fresc.2023.1170256

**Published:** 2023-03-17

**Authors:** Frontiers Production Office

**Affiliations:** Frontiers Media SA, Lausanne, Switzerland

**Keywords:** word learning, shared storybook reading, developmental language disorder (DLD), Dynamic assessment (DA), Diagnostic accuracy, bilingualism, Children

An Erratum on A new dynamic word learning task to diagnose language disorder in French-speaking monolingual and bilingual children By Matrat M, Delage H and Kehoe M. (2023) Front. Rehabilit. Sci. 3:1095023. doi: 10.3389/fresc.2022.1095023

An omission to the funding section of the original article was made in error. The following sentence has been added: “Open access funding was provided by the University of Geneva”.

The original version of this article has been updated.

