# Supplementary material for: A new dynamic word learning task to diagnose language disorder in French-speaking monolingual and bilingual children
Source: Front Rehabil Sci. 2023 Jan 30;3:1095023. doi: 10.3389/fresc.2022.1095023 (PMC9922851; doi:10.3389/fresc.2022.1095023)
Supplement: Supplementary file 1 [file Datasheet1.docx]

**Appendix**

**Appendix 1. Script**

1. This is the story of Leo who decides to go on an adventure. He puts his (*pointing*) /**fuk**/ and his (*pointing*) /**moze**/ in his bag and leaves his house.

2. Leo gets into his (*pointing*) /**pitɛl**/. He flies, his /**pitɛl**/ goes very fast!

3. Leo is tired, he decides to stop. He puts down his /**pitɛl**/ [Did you see his (*pointing*) /**pitɛl**/? It's flying! Can you repeat /**pitɛl**/? Yes, good.] Leo puts his /**moze**/ on his head to jump on the ground.

4. He sees a /**klan**/ next to the tree. The /**klan**/ likes chocolate a lot! [Do you like chocolate too, just like the (*pointing*) /**klan**/?]

5. Leo is hungry. He goes to get his /**fuk**/ from the /**pitɛl**/.

6. He turns on his /**fuk**/ to find some sweets. Leo eats the sweets. Hm, they are delicious! Leo turns off his /**fuk**/. [I would love to have (*pointing*) a /**fuk**/ to find some sweets, how about you?]

7. Leo puts his /**fuk**/ and /**moze**/ back in his bag. [Look at (*pointing*) his /**moze**/! Can you repeat /**moze**/? Yes, good.]

8. Leo yawns, climbs into his /**pitɛl**/ and puts his bag down. Then he falls asleep next to his /**pitɛl**/. [Have you ever seen a (*pointing*) /**pitɛl**/?]

9. When he wakes up, Leo bangs his head against his /**pitɛl**/. He climbs into his /**pitɛl**/.Oh no! His bag is gone! The /**klan**/ watches Leo search everywhere for his bag.

10. Suddenly, Leo looks up and sees his bag hanging from a tree. The /**klan**/ also sees the bag!

11. Leo climbs up the tree and discovers who has stolen his bag: a bird. The bird is holding the /**moze**/ in its beak. The /**fuk**/ is hanging on a branch. [Can you see (*pointing*) the /**fuk**/ there? Can you repeat /**fuk**/? Yes, good.]

12. Seeing Leo, the bird flies away and drops the /**moze**/. Oh no, Leo can't catch the /**moze**/!

13. The /**moze**/ falls next to the /**klan**/. The /**klan**/ is surprised. [Look (*pointing*) at the /**klan**/ there! Can you repeat /**klan**/? Yes, good.]

14. Leo grabs his /**fuk**/ and his bag. He holds his /**fuk**/ tightly so he doesn't drop it. Then he climbs down the tree.

15. Worried, he picks up his /**moze**/, puts it on his head and makes a big jump. Phew, the /**moze**/ still works! [Would you like to jump with (*pointing*) the /**moze**/ just like Leo?]

16. The /**klan**/ comes up to him and gives a little scream. The /**klan**/ also wants to try the {*Completion* + *pointing*: It's [a /**moze**/,] a hat that lets you jump! Can you repeat /**moze**/? Yes, bravo.} Gee, his head is too small to wear it!

17. Leo wants to give the /**klan**/ some sweets. So he takes out his /**fuk**/.

18. It's time to go home. Leo goes back to his /**pitɛl**/. Leo says goodbye to the {*Completion* + *pointing*: It's a /**klan**/, an animal that likes chocolate. Can you repeat /**klan**/? Yes, bravo.}

19. Leo starts his {*Completion* + *pointing*: It's a /**pitɛl**/ a car that is used for flying. Can you repeat /**pitɛl**/? Yes bravo.} He has a lot of driving to do! He flies fast to get home. In the distance, the /**klan**/ is eating chocolate.

20. When he gets home, he parks his /**pitɛl**/. Going up the stairs, Leo drops his {*Completion* + *pointing*: It's a /**fuk**/, a machine used to find sweets. Can you repeat /**fuk**/? Yes bravo.} on the floor.

21. He goes home, turns off his /**fuk**/ and puts his /**moze**/ on the table.

22. He can finally fall asleep quietly, with stars in his eyes, ready to travel even further into the land of dreams.

Legend: *[Comment] {Completion}*

**Appendix 2. Examples of pictures from the storybook**


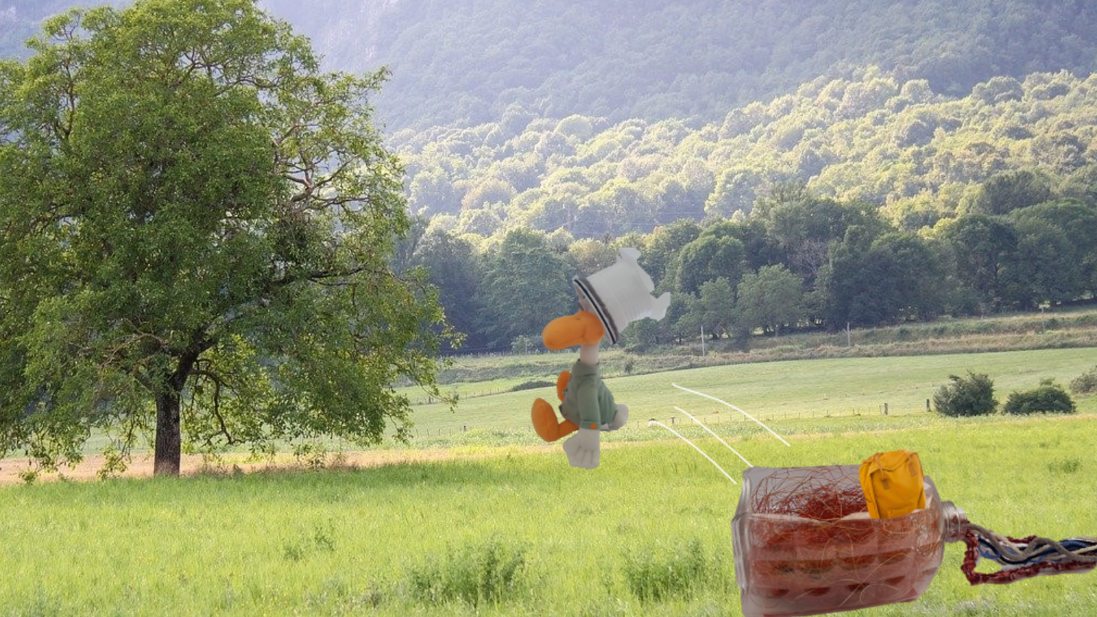

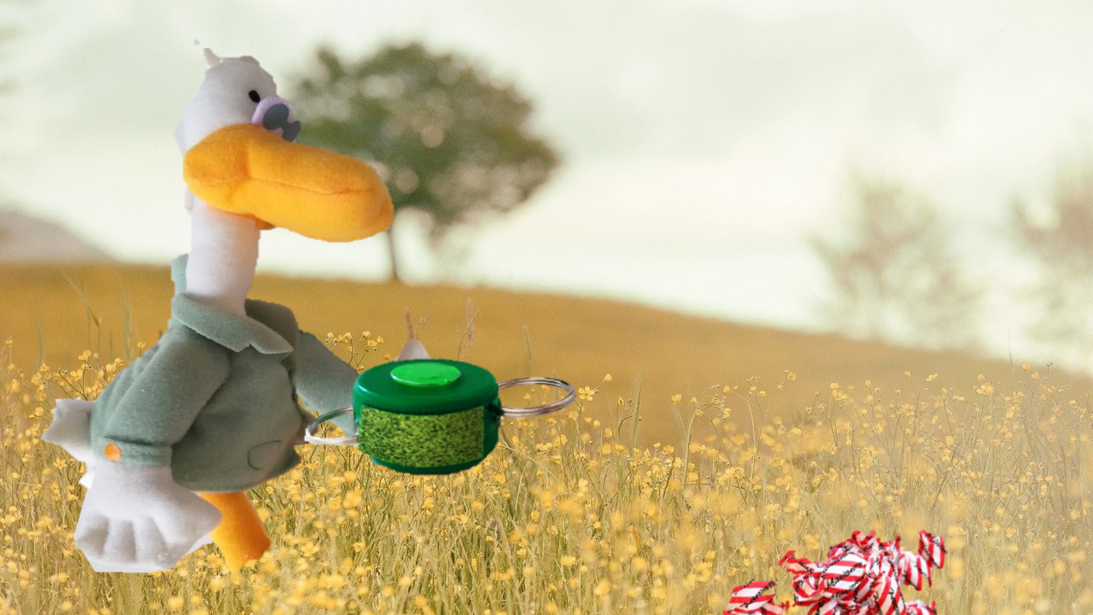

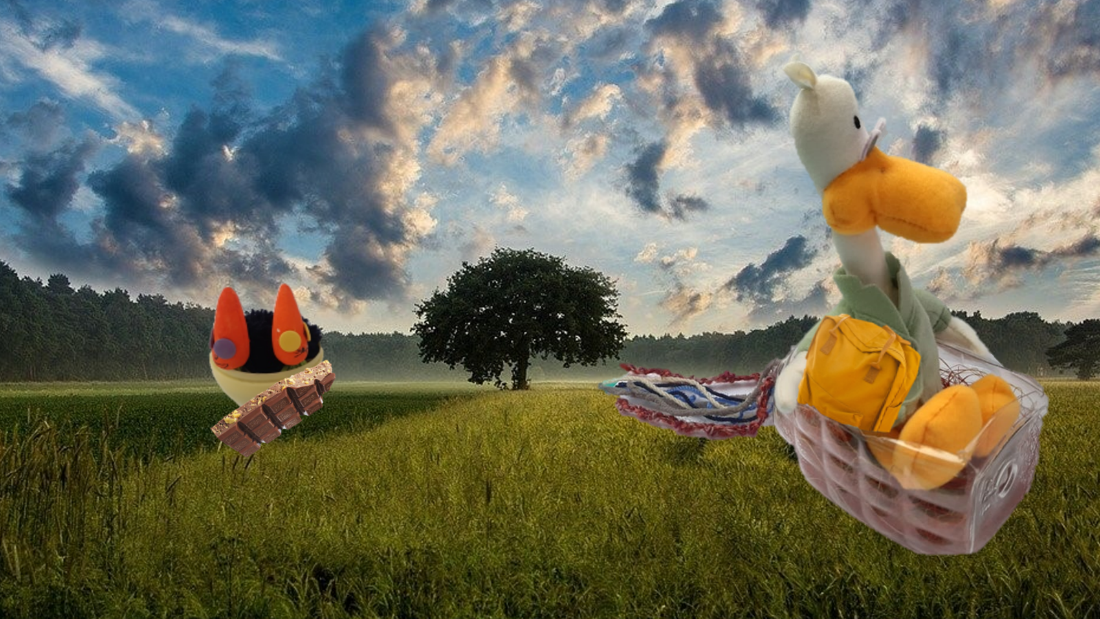


Image 3. Image 6. Image 19.

**Appendix 3. Results on generalized linear mixed models.**

3.1. Results on generalized linear mixed model without interactions for Phonological Prompts.

|  | **Reference** | **Estimate** | **Std. error** | **Z value** | **Pr(>\|z\|)** |
| --- | --- | --- | --- | --- | --- |
| **(Intercept)** |  | 0.69860 | 0.20680 | 3.378 | 0.00073 *** |
| **Version** | 2 | -0.46925 | 0.19621 | -2.392 | 0.01678 * |
| **Age** |  | 0.52024 | 0.10557 | 4.928 | 8.31e-07 *** |
| **Gender** | boys | 0.38130 | 0.20862 | 1.828 | 0.06760 . |
| **Education of parent** |  | 0.12481 | 0.10872 | 1.148 | 0.25098 |
| **Time** | delayed | -0.50750 | 0.08191 | -6.196 | 5.80e-10 *** |
| **Developmental group (DG)** | DLD | -0.96298 | 0.22206 | -4.337 | 1.45e-05 *** |
| **Linguistic status (LS)** | bilingual | -0.32046 | 0.18300 | -1.751 | 0.07991 . |

*Education of Parent = number of years of tertiary education; DLD = Developmental Language Disorder group;*

*p = < .10 (*); < .05 * ; < .01 **; < .001 ****

3.2. Results on generalized linear mixed model without interactions for Semantic Prompts.

|  | **Reference** | **Estimate** | **Std. error** | **Z value** | **Pr(>\|z\|)** |
| --- | --- | --- | --- | --- | --- |
| **(Intercept)** |  | 1.08397 | 0.31726 | 3.417 | 0.000634 *** |
| **Version** | 2 | -0.43387 | 0.22257 | -1.949 | 0.05125 . |
| **Age** |  | 0.54649 | 0.11802 | 4.631 | 3.65e-06 *** |
| **Gender** | boys | 0.49247 | 0.23450 | 2.100 | 0.03573 * |
| **Education of parent** |  | 0.14762 | 0.12675 | 1.165 | 0.24418 |
| **Time** | delayed | 0.98444 | 0.08005 | 12.298 | < 2e-16 *** |
| **Developmental group (DG)** | DLD | -0.70984 | 0.24898 | -2.851 | 0.00436 ** |
| **Linguistic status (LS)** | bilingual | 0.33431 | 0.20869 | 1.602 | 0.10917 |

*Education of Parent = number of years of tertiary education; DLD = Developmental Language Disorder group;*

*p = < .10 (*); < .05 * ; < .01 **; < .001 ****

3.3. Results on generalized linear mixed model for the category score.

|  | **Reference** | **Estimate** | **Std. error** | **Z value** | **Pr(>\|z\|)** |
| --- | --- | --- | --- | --- | --- |
| **(Intercept)** |  | -0.1761 | 0.4282 | -0.411 | 0.680850 |
| **Version** | 2 | -0.70124 | 0.26876 | -2.609 | 0.009076 ** |
| **Age** |  | 0.55050 | 0.14178 | 3.883 | 0.000103 *** |
| **Gender** | boys | 0.76859 | 0.28193 | 2.726 | 0.006408 ** |
| **Education of parent** |  | 0.19531 | 0.15187 | 1.286 | 0.198445 |
| **Time** | delayed | 1.41001 | 0.12919 | 10.914 | < 2e-16 *** |
| **Developmental group (DG)** | DLD | 0.3714 | 0.4040 | 0.919 | 0.357947 |
| **Linguistic status (LS)** | bilingual | 0.89373 | 0.29750 | 3.004 | 0.002663 ** |
| **Time x DG** | delayed/DLD | -0.46865 | 0.22025 | -2.128 | 0.033351 * |
| **DG x LS** | DLD/bilingual | -1.58321 | 0.53929 | -2.936 | 0.003328 ** |

*Education of Parent = number of years of tertiary education; DLD = Developmental Language Disorder group;*

*p = < .10 (*); < .05 * ; < .01 **; < .001 ****

3.4. Results on generalized linear mixed model for the definition score.

|  | **Reference** | **Estimate** | **Std. error** | **Z value** | **Pr(>\|z\|)** |
| --- | --- | --- | --- | --- | --- |
| **(Intercept)** |  | 3.8146 | 0.6670 | 5.719 | 1.07e-08 *** |
| **Version** | 2 | -0.6159 | 0.5782 | -1.065 | 0.287 |
| **Age (month)** |  | 1.6968 | 0.3492 | 4.860 | 1.18e-06 *** |
| **Gender** | boys | 0.5852 | 0.6209 | 0.942 | 0.346 |
| **Education of parent** |  | 0.3641 | 0.3571 | 1.020 | 0.30 |
| **Time** | delayed | 1.1676 | 0.1831 | 6.378 | 1.80e-10 *** |
| **Developmental group (DG)** | DLD | -1.9403 | 0.6741 | -2.878 | 0.004 ** |
| **Linguistic status (LS)** | bilingual | 0.7217 | 0.5550 | 1.300 | 0.193 |

*Education of Parent = number of years of tertiary education; DLD = Developmental Language Disorder group;*

*p = < .10 (*); < .05 * ; < .01 **; < .001 ****
